# Genomic properties of a *Bartonella quintana* strain from Japanese macaque (*Macaca fuscata*) revealed by genome comparison with human and rhesus macaque strains

**DOI:** 10.1038/s41598-024-61782-0

**Published:** 2024-05-13

**Authors:** Shingo Sato, Emu Nishioka, Hidenori Kabeya, Soichi Maruyama

**Affiliations:** 1 Laboratory of Veterinary Public Health, Department of Veterinary Medicine, College of Bioresource Sciences, Nihon University, 1866 Kameino, Fujisawa, Kanagawa 252-0880 Japan; 2 Laboratory of Veterinary Food Hygiene, Department of Veterinary Medicine, College of Bioresource Sciences, Nihon University, 1866 Kameino, Fujisawa, Kanagawa 252-0880 Japan

**Keywords:** Bacterial genetics, Bacterial evolution, Bacterial genomics

## Abstract

*Bartonella quintana*, the causative agent of trench fever, is an intracellular bacterium that infects human erythrocytes and vascular endothelial cells. For many years, humans were considered the only natural hosts for *B. quintana*; however, it was recently discovered that wild Japanese macaques (*Macaca fuscata*) also serve as hosts for *B. quintana*. To elucidate the genetic characteristics of the *B. quintana* strain MF1-1 isolated from a Japanese macaque, we determined the complete genome sequence of the strain and compared it with those of strain Toulouse from a human and strain RM-11 from a rhesus macaque. General genomic features and orthologous gene cluster profiles are similar among the three strains, and strain MF1-1 is genetically closer to strain RM-11 than strain Toulouse based on the average nucleotide identity values; however, a significant inversion of approximately 0.68 Mb was detected in the chromosome of strain MF1-1. Moreover, the Japanese macaque strains lacked the *bepA* gene, which is responsible for anti-apoptotic function, and the *trwL2*, *trwL4*, and *trwL6* genes, which may be involved in adhesion to erythrocytes of rhesus macaque and human. These features likely represent the genomic traits acquired by Japanese macaque strains in their host-associated evolution.

## Introduction

*Bartonella quintana* is the causative agent of trench fever and is transmitted via human lice (*Pediculus humanus*) among humans. Patients with trench fever show several clinical symptoms, including headache, bacteremia, dizziness, persistent pain in both tibias, and relapsing fever at 5-day intervals^1^. Clinical symptoms are more severe in HIV-positive patients with *B. quintana* infection, where endocarditis with cardiac vegetation and bacillary angiomatosis are often observed as serious complications^2^. Bacillary angiomatosis is characterized by vascular tumors comprising tumorigenic human endothelial cells and occurs in systemic cutaneous regions and visceral organs, such as the liver, spleen, bone marrow, and eyes^3,4^.

An initial step in *Bartonella* infection is the direct attachment to the extracellular matrix around vascular endothelial cells and the cell membrane^5^. To achieve this step, *B. quintana* mainly uses variably expressed outer membrane proteins (Vomps), which are classified as trimeric autotransporter adhesins, consist of four subtypes (A–D)^6^. Therefore, the Vomps are essential for the initial stage of *B. quintana* infection. After adhering to the vascular endothelial cells, *Bartonella* effector proteins (Beps) are translocated into the endothelial cells via the VirB/D4 type IV secretion system (T4SS) and exert multiple physiological effects^7^. Six *bep* genes (*bepA1*, *bepA2*, *bepC*, *bepE*, *bepF1*, and *bepF2*) are tandemly encoded in the *virB/D4* operon of *B. quintana*, and all genes except *bepA1* retain a Bep-intracellular delivery (BID) domain at their C-terminus^8^. Notably, the BID domain of *bepA2* has an anti-apoptotic function on infected endothelial cells by elevating the cellular cAMP concentration^9^. Consequently, protecting infected cells from apoptosis is involved in the formation of vascular tumors. A recent study demonstrated that a new proangiogenic autotransporter, BafA, can act as an agonist of the vascular endothelial growth factor receptor 2^10^. These findings suggest that the development of bacillary angiomatosis is promoted not only by the BID domain of *bepA2* but also by BafA.

After the infection of vascular endothelial cells by *B. quintana*, the bacteria are shed into the host bloodstream, and some of the bacterial population adheres to erythrocytes via the Trw T4SS. This adhesion ability of the Trw T4SS is a critical determinant of host specificity for *Bartonella* species^11^. TrwJ and TrwL, which constitute pilus-like structures in the Trw T4SS, have been recognized as key factors in host specificity determination^11^. The gene copy number of these two Trw proteins varies among *Bartonella* species; two copies of *trwJ* and eight copies of *trwL* are encoded in a previously sequenced *B. quintana* genome^12^. It has also been speculated that *B. quintana* actively invades human erythrocytes via IalB^13^ after attaching to the erythrocytes in a manner similar to *B. bacilliformis*, the causative agent of Carrion’s disease. HbpA, a member of the hemin-binding protein family (HbpA to E)^14,15^, has also been shown to localize to the outer membrane of *B. quintana* and to acquire the hemin within human erythrocytes as a nutrient source.

For many years, humans were considered the only natural host of *B. quintana*. However, *B. quintana* was recently detected in cynomolgus and rhesus macaques bred in primate research institutes in China^16,17^. Based on this finding, we investigated the prevalence of *B. quintana* in wild populations of Japanese macaques^18^ and found that 13.3% (6/45) of the wild-caught macaques were positive for *B. quintana* bacteremia without any clinical symptoms. *B. quintana* strains were grouped according to host species such as humans (STs 1–7 and 23–27)^19,20^, cynomolgus macaques (STs 15–21), rhesus macaques (STs 8–14)^17^, and Japanese macaques (ST22)^18^ by multi-locus sequence typing (MLST). Even though the same typing tool was applied to these *B. quintana* strains, the Japanese macaque strains presented considerably a low genetic diversity compared to other human and macaque strains. It is interpreted that there was not enough evolutionary time for the Japanese macaque strains to accumulate many mutations in the genome. Therefore, we speculated that the Japanese macaque strains might be “newcomers” in the history of adaptive evolution for *B. quintana*.

In this study, we determined the complete genome sequence of *B. quintana* strain MF1-1 from a Japanese macaque and compared it with previously determined complete genomes of strains RM-11 (from a rhesus macaque) and Toulouse (from a human) to elucidate the genomic properties including virulence genes of the Japanese macaque strain. In addition, other *B. quintana* strains from Japanese macaques were analyzed to explore the genomic structures of the *virB/D4* and *trw* operons specific to the strains from Japanese macaque.

## Methods

### Bacterial strain and whole-genome sequencing

The bacterial strain used for whole-genome sequencing was *Bartonella quintana* strain MF1-1, which had been isolated from a blood of a Japanese macaque (ID# MF1) in a previous study^18^. The macaque MF1 had been captured in Wakayama Prefecture, Japan following the Wildlife Protection, Control, and Hunting Management Act in Japan, and the related data has been published ^18^. The strain was cultured on 5% chocolate solid agar plates containing 5% rabbit blood at 35 °C under 5% CO_2_ for 10 days.

Whole-genome sequencing was performed using a combined approach with long-read and short-read sequencing technologies. A PacBio RS II sequencer (Pacific Biosciences, Menlo Park, CA, USA) and a HiSeq2500 sequencer (Illumina, San Diego, CA, USA) were used for long-read and short-read sequencing, respectively. Genomic DNA was extracted from the MF1-1 cells grown on chocolate solid agar plates^18^ using a MagAttract HMW DNA Kit (Qiagen, Venlo, The Netherlands) according to the manufacturer’s instructions. A single-molecule real-time bell library was constructed from the fragmented genomic DNA using a SMRTbell Template Prep Kit (Pacific Biosciences, Menlo Park, CA, USA), and then sequenced using the PacBio RSII sequencer with P6-C4 chemistry. 16,395 long-reads were generated from the sequencer, and the total read length were 214,637,280 bp with a sequencing depth of × 135 coverage. The genomic DNA of strain MF1-1 was also extracted using InstaGene Matrix (Bio-Rad, Hercules, CA, USA), and used to prepare a sequencing library for Illumina sequencing using TruSeq Nano DNA Library Prep Kit (Illumina, San Diego, CA, USA) according to the manufacturer’s instructions. The DNA library was sequenced using a HiSeq2500 sequencer to obtain paired-end sequences (2 × 100 bp). 11,851,882 short-reads were generated from the sequencer, and the total read length were 1,196,924,269 bp with a sequencing depth of × 743 coverage.

### **De novo assembly with long-read sequences and polishing with short-read sequences**

Long-read sequences were de novo assembled using the assembler program “CANU v. 1.0.6”^21^ implemented in Maser^22^, a web-based analysis environment specifically designed for next-generation sequencers (NGS) data, to construct a draft genome sequence of strain MF1-1. Subsequently, short-read sequences were used to polish the draft genome sequence without any gaps. Before the polishing, the short-reads were trimmed and filtered according to the read length and quality (minimum length 100 bp, quality score 0.05) in the CLC Genomic workbench v.8.5.1 (Qiagen, Venlo, The Netherlands) and then mapped in the draft genome sequence using the NGS core tool “Map Reads to Reference” in the CLC Genomic workbench^23^. Mapping parameters in this study are as follows: mismatch cost = 1, insertion cost = 2, deletion cost = 2, auto-detected paired distance = yes, length fraction = 0.8, and similarity fraction of 0.9. A consensus sequence was extracted from the mapped genome sequence, then sequencing errors were detected by an analysis tool “Basic Variant Detection” in the same NGS software, followed by manual curation to construct the complete genome sequence.

### Genome annotation and construction of a circular genome map

The complete genome sequence was annotated using a web-based annotation pipeline, the DDBJ fast annotation and submission tool (DFAST)^24^ provided by the National Institute of Genetics, Japan. To estimate the number of protein-coding genes (PCGs), rRNAs, and tRNAs, we chose a default setting in DFAST: MetaGeneAnnotator for PCGs, Barrnap^25^ for rRNAs, and tRNAscan-SE^26^ for tRNAs. A pre-formatted GenBank file of strain MF1-1 was outputted from DFAST and was deposited in the International Nucleotide Sequence Database (INSD) cooperatively managed by National Center for Biotechnology Information (NCBI), DDBJ center, and EMBL-EBI.

The circular genome map of MF1-1 was constructed using the CGView server30 (https://proksee.ca/)^27^, a visualization tool for bacterial genomes. The GC skew was calculated based on sliding windows of 10 kb and a step size of 0.1 kb in the complete genome sequence. The replication origin of the chromosomal genome (*oriC* motif) was predicted by Ori-Finder2 program^28^. The terminus region was visually detected with reference to the *dif* motif^29^.

### Average nucleotide identity analysis

Nucleotide sequence identities at whole-genome levels were assessed by pairwise average nucleotide identity (ANI) values calculated with BLAST^30,31^. The genome sequence of strain MF1-1 was compared with those of strains Toulouse, RM-11, six human strains, a human louse strain, and type strains of other known 14 *Bartonella* species: *B. henselae* strain Houston-1^ T^, *B. koehlerae* strain C-29^ T^, *B. alsatica* strain IBS382^T^, *B. birtlesii* strain IBS325^T^, *B. doshiae* strain R-18^ T^, *B. grahamii* strain V2^T^, *B. florencae* strain R4^T^, *B. elizabethae* strain F9251^T^, *B. bovis* strain 91-4^ T^, *B. bacilliformis* strain KC583^T^, *B. rochalimae* strain BMGH^T^, *B. ancashensis* strain 20.00^ T^, *B. tamiae* strain Th239^T^, and *B. apis* strain PEB0122^T^.

### Whole-genome sequence data

*B. quintana* strain Toulouse from a patient with bacillary angiomatosis in France and strain RM-11 from a rhesus monkey in a primate center in China were selected as representatives of complete genome sequence data for comparative genomic analyses with strain MF1-1. Additionally, strains CO20_0256, CO20_0257, CO20_0297, CO20_0321, and CO21_0024 from patients in the USA and strains G1712 and G1713 from a human and a human louse in Senegal, respectively, were added to the study. To unify the genome annotation level of the sequence data, the Reference Sequence (RefSeq) database, managed by the NCBI, was used in the present analysis. The RefSeq accession numbers of the used strains were NZ_AP019773 for strain MF1-1, NC_005955 for strain Toulouse, NC_018533 for strain RM-11, NZ_CP076602 for strain CO20_0256, NZ_CP076601 for strain CO20_0257, NZ_CP076600 for strain CO20_0297, NZ_CP076599 for strain CO20_0321, NZ_CP076598 for strain CO21_0024, NZ_CP091505 for strain G1712, and NZ_CP091504 for strain G1713.

### Comparative genomic analyses

The deduced amino-acid sequences were translated from the PCGs of strains MF1-1, RM-11, and Toulouse, and submitted to eggNOG-mapper v.2, a rapid functional annotation tool for large sets of amino-acid sequences^32^. Next, all the amino-acid sequences were functionally classified based on the Clusters of Orthologous Genes (COGs) database^33^ updated in 2020 (available at https://www.ncbi.nlm.nih.gov/research/cog/), and the COG profiles were compared among the three strains.

The core-genome and pan-genome of the three *B. quintana* strains were investigated using EDGAR 3.0, a comprehensive microbial genome comparative analysis tool^34^. This calculation was performed based on the reciprocal best BLAST hits and the BLAST score ratio value approach. When a query had no orthologs in the other two strains, the gene was defined as a singleton (i.e., a strain-specific gene). If paralogous genes were detected in the genomes, one gene per paralog was used for the analysis.

To visualize genomic structural variations, such as large-scale insertions, deletions, and inversions, the collinearities of the genome sequences were analyzed by dot plot analysis using the IMC genomics software (in silico biology, Inc., Kanagawa Prefecture, Japan).

### Comparison of the virulence gene repertoires

To evaluate the differences in potential virulence between strains MF1-1, RM-11, and Toulouse at the genome level, we first screened for the presence/absence of virulence genes encoded in the genomes using the virulence factor database^35^. Subsequently, the detected virulence genes were compiled for each strain with reference to the results of the core-genome analysis. Gene synteny of the *virB/D4* and *trw* operons between the three *B. quintana* strains was analyzed using the IMC genomics software (in silico biology, Inc., Kanagawa Prefecture, Japan). Additionally, the other seven strains (CO20_0256, CO20_0257, CO20_0297, CO20_0321, CO21_0024, G1712, and G1713) were used to further investigate the whole structures of the *virB/D4* and *trw* operons. The orthologous genes in the operons were aligned and their nucleotide sequence identities were calculated using GENETYX v.15 (GENETYX Corp., Tokyo, Japan).

### PCR detection and DNA sequencing of the *bepA* and *trwL* loci

We performed *bepA*-specific PCR to determine whether the *bepA* locus (composed of *bepA1* and *bepA2*) is present in the *virB/D4* operon of other *B. quintana* strains (MF3-1, MF10-1, MF11-1, MF19-1, and MF34-1) from Japanese macaques^18^. In addition, *trwL*-specific PCR was performed to determine whether the *trwL* locus (composed of *trwL1* to *trwL8*) is present in the *trw* operon of the five Japanese macaque strains. TaKaRa LA Taq Hot Start Version (TaKaRa Bio Inc., Shiga, Japan) was used as DNA polymerase to amplify both loci by the PCRs. Genomic DNA from *B. quintana* strain Fuller^T^ and nuclease-free distilled water were used as positive and negative controls, respectively, for both PCRs. The PCR amplicons were purified using a Wizard SV Gel and PCR Clean-Up System (Promega Corporation, Madison, WI, USA), followed by direct DNA sequencing using the Genetic Analyzer models 3130xl or 3730xl (Thermo Fisher Scientific, Waltham, MA, USA). The PCR primers and conditions used in this study are described in Supplementary Tables S1 and S2, respectively.

## Results and discussion

### General genomic features of strain MF1-1

The complete genome sequence of strain MF1-1 was constructed from a combined assembly using long- and short-read sequencing data. The genome size of strain MF1-1 was 1,588,683 bp in sequence length, and its G + C content in the genome was 38.8% (Table 1).

**Table 1:** General genomic features of *B. quintana* strain MF1-1 compared with strains RM-11 and Toulouse.

Strain name for *B. quintana*	MF1-1	RM-11	Toulouse
Host name (Scientific name)	Japanese macaque (*Macaca fuscata*)	Rhesus macaque (*Macaca mulatta*)	Human (*Homo sapiens*)
Chromosomal genome size (bp)	1,588,683	1,587,646	1,581,384
Average G + C content (%)	38.8	38.8	38.8
Number of rRNAs (5S, 16S, and 23S)	6 (2, 2, 2)	6 (2, 2, 2)	6 (2, 2, 2)
Number of tRNAs	42	42	42
Number of protein-coding genes	1179	1149	1176
Average gene length (bp)	978	975	975
Gene length/total genome length (%)	72.6	70.6	72.6

**Figure 1:**
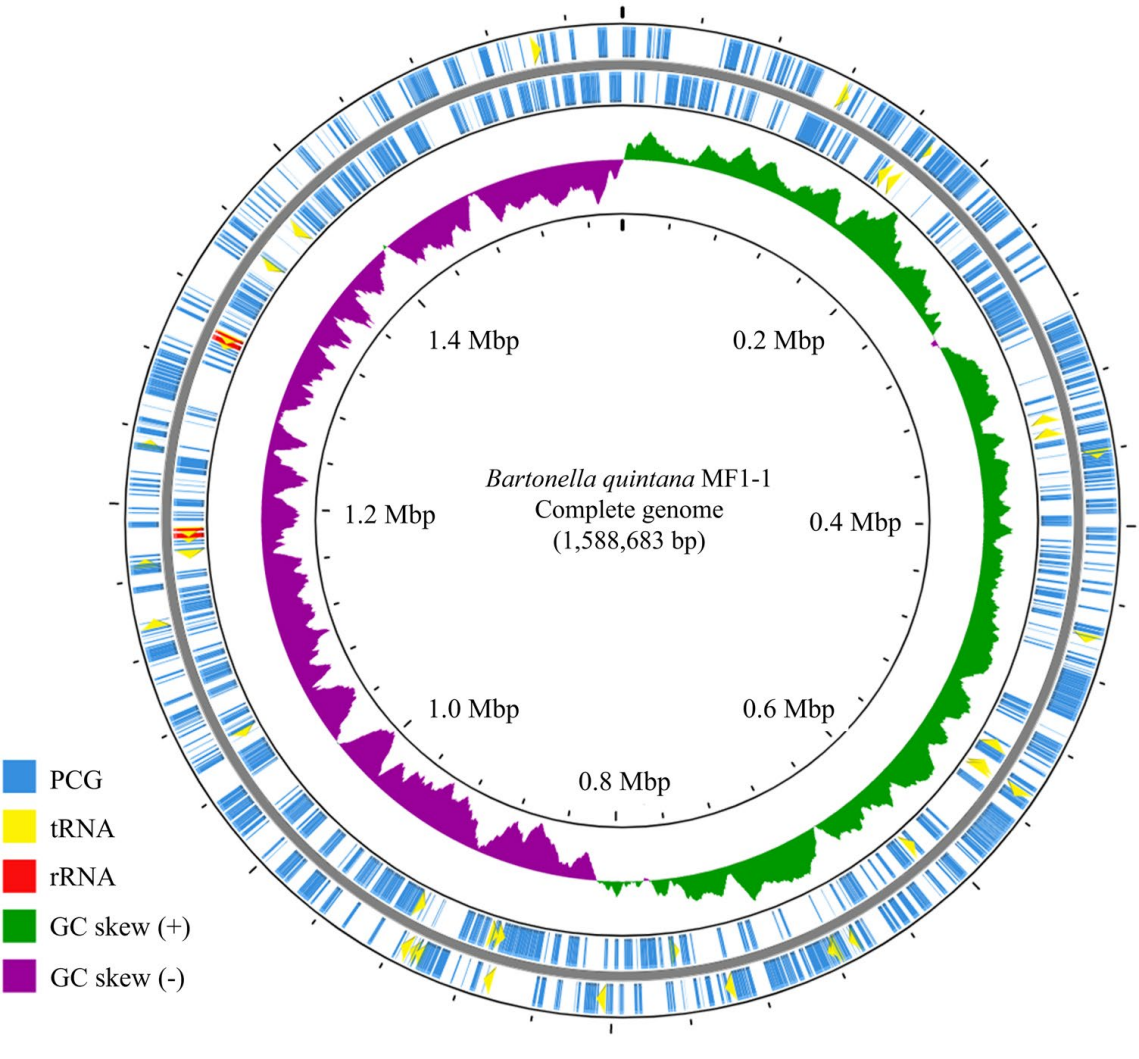
Circular genome map of *Bartonella quintana* strain MF1-1. The circles indicate the following features: outermost circle, PCGs transcribed clockwise on the forward strand; second circle, PCGs transcribed anticlockwise on the reverse strand; third circle, GC skew in a sliding window of 10 kb and step size of 0.1 kb; innermost circle, DNA base position.

Plasmids are generally absent in most *Bartonella* species, except *B. schoenbuchensis*^36^, *B. grahamii*^37^, and *B. tribocorum*^38^. In this study, strain MF1-1 was found to have a single circular genome without any plasmids (Fig. 1). Similar to strain MF1-1, no plasmid has been reported in *B. quintana* strain RM-11 from a rhesus macaque and strain Toulouse from a human, suggesting that *B. quintana* contains no plasmids regardless of the host origin. The switching points of the GC bias were symmetrical at equidistant positions in the genome of strain MF1-1 (Fig. 1). The putative replication origin (*oriC* motif) and terminus region (*dif* motif) in the genome were observed at the corresponding positions for the two switches above (Supplementary Fig. S1). In bacteria, the chromosomal replication origin and terminus region are usually located at the switching points of genomic GC bias. Thus, these data support that the complete genome map of strain MF1-1 was correctly generated without misassembly of the NGS sequencing reads.

Genome annotation predicted 1,179 PCGs from the complete genome of strain MF1-1 (Table 1). The numbers of tRNAs and rRNAs were estimated to be 42 and 6, respectively. The rRNA operon comprising 5S rRNA, 16S rRNA, and 23S rRNA was present in two copies in the genome, which agrees with previous reports^38,39^. The genomic features of strains RM-11 and Toulouse were almost identical to the relevant data from strain MF1-1, even though the compared strains were derived from a rhesus macaque and a human. Therefore, the basic properties of the *B. quintana* genome are likely to be common regardless of the host species.

### ANI analysis with strain MF1-1, the other *B. quintana* strains, and other known *Bartonella* species

**Figure 2:**
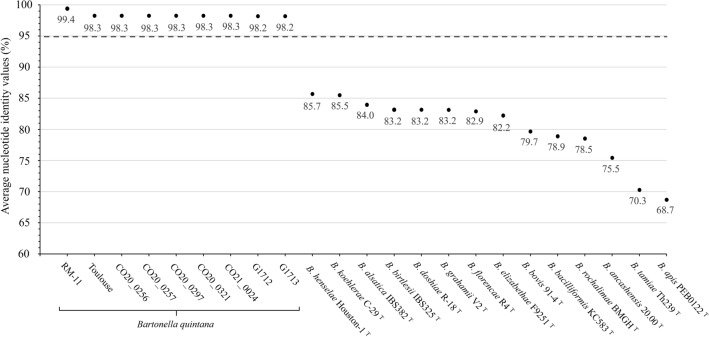
Average nucleotide identity values (%) between *B. quintana* strain MF1-1 and other *B. quintana* strains and type strains of other known *Bartonella* species. The dashed line represents a 95% cut-off value to discriminate *Bartonella* species.

An ANI value of 95% has been defined as the cut-off value to discriminate bacterial species according to a previous report^40^. The ANI values between strain MF1-1 and type strains of other 14 known *Bartonella* species ranged from 68.7% (vs. *B. apis*) to 85.7% (vs. *B. henselae*), which were below the cut-off value (Fig. 2). In contrast, the ANI values between strain MF1-1 and other *B. quintana* strains ranged from 98.2% to 99.4%, which exceeded the cut-off value. When comparing the *B. quintana* strains by pairwise comparison, strain MF1-1 showed the highest ANI value (99.4%) with strain RM-11 (Supplementary Fig. S2), suggesting a genetically high similarity between macaque-derived strains.

### Comparative genomic analyses

**Figure 3:**
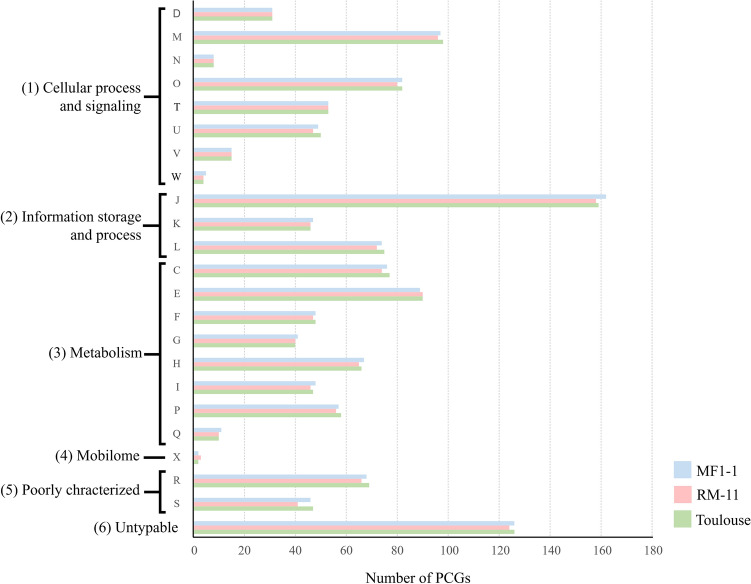
COGs classification of the PCGs in *B. quintana* strains MF1-1, RM-11, and Toulouse. The PCGs of the three *B. quintana* strains were classified into each COG class, and the number of PCGs is indicated by colored bars: strain MF1-1 (blue), strain RM-11 (red), and strain Toulouse (green). (1) Cellular process and signaling: D, cell cycle control, cell division, and chromosome partitioning; M, cell wall/membrane/envelope biogenesis; N, cell motility; O, posttranslational modification, protein turnover, and chaperones; T, signal transduction mechanisms; U, intracellular trafficking, secretion, and vesicular transport; V, defense mechanisms; W, extracellular structures. (2) Information storage and process: J, translation, ribosomal structure, and biogenesis; K, transcription; L, replication, recombination, and repair. (3) Metabolism: C, energy production and conversion; E, amino-acid transport and metabolism; F, nucleotide transport and metabolism; G, carbohydrate transport and metabolism; H, coenzyme transport and metabolism; I, lipid transport and metabolism; P, inorganic ion transport and metabolism; Q, secondary metabolites biosynthesis, transport, and catabolism. (4) Mobilome: prophages and transposons. (5) Poorly characterized: R, general function prediction only; S, function unknown. (6) Untypable: PCGs untyped in COG classification.

COG classification was applied to infer the biological functions of the PCGs identified in the three *B. quintana* strains (Fig. 3). The COG profiles of the three strains were very similar in each of the major categories, “Cell processing and signaling,” “Information storage and process,” “Metabolism,” and “Mobilome”. This result indicates that the molecular mechanisms involved in survival and proliferation may be similar regardless of *B. quintana* strains. Furthermore, we examined the core-genome genes shared by the three strains and identified 1,111 PCGs (Supplementary Fig. S3 and Supplementary Table S3), which corresponds to 91.0% of the pan-genome genes (1,221 PCGs) of the three *B. quintana* genomes. In a previous genomic analysis of four *B. elizabethae* strains^41^, 96.6% (2,064 PCGs) of the pan-genome genes (2,137 PCGs) were identified as core-genome genes. The present and previous data support the idea that a large proportion of the pan-genome genes are conserved as core-genome genes among different strains of the same *Bartonella* species. Although Japanese macaques have been classified into two major haplogroups based on their mitochondrial DNA sequences^42^, all *B. quintana* strains from Japanese macaques were classified into a single ST (ST22) by MLST targeting nine housekeeping genes^18^. More distinguishable typing tools, such as core-genome MLST and/or core-genome single-nucleotide polymorphism, are necessary to elucidate the genetic diversity of *B. quintana* strains prevalent in Japanese macaques. Consequently, the core-genome genes identified in this study will be available for developing these new genome-typing tools for *B. quintana*.

**Figure 4:**
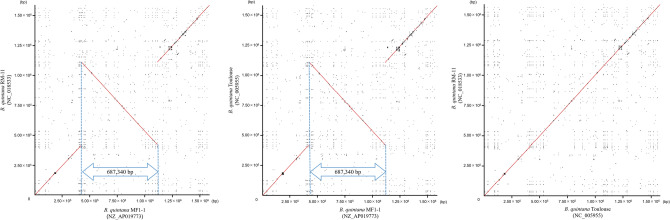
Genome-wide collinearity among the *B. quintana* strains MF1-1, RM-11, and Toulouse by dot plot analysis. The complete genomes of strains MF1-1, RM-11, and Toulouse were compared. Counting from the highest sequence identities, 10^5^ dots are preferentially displayed in the plot. Red and black dots denote more than or equal to 95% sequence identities and less than 95% sequence identities, respectively.

**Figure 5:**
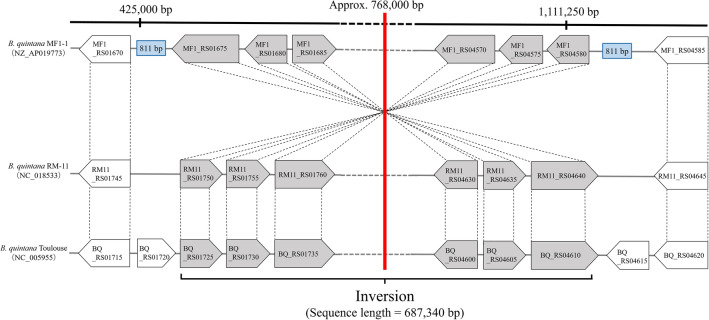
Expanded comparison view of genomic rearrangement among the *B. quintana* strains MF1-1, RM-11, and Toulouse. Orthologous genes are connected by dashed lines, and each gene is presented by a locus tag number. A red central line indicates a symmetrical axis of the inversion, and an approximate position is described at the top of the red line. Blue boxes indicate long homologous sequences at the two ends of the inversion; the sequences on the left and right sides are located at 425,037–425,847 bp and 1,113,749–1,114,559 bp, respectively.

Dot plot analysis of the RM-11 and Toulouse genomes showed genome-wide sequence collinearity without any detectable large structural variations, indicating that these two strains have similar genomic structures. In contrast, an inversion of an approximately 0.68 Mb segment was observed in the MF1-1 chromosome compared with the other two strains (Fig. 4). A symmetric axis of the inversion was located at about 768,000 bp in the MF1-1 chromosome, which is close to the replication terminus region between 814,766 and 814,793 bp. Additionally, long homologous sequences (each length = 811 bp) were also observed at the two ends of the inversion (Fig. 5). The MF1-1 chromosome encodes a recombinase gene often found in bacteria, *recA* (locus tag number = MF1_RS02330); this inversion might have occurred by homologous recombination based on the homologous sequences. We should determine the complete genome sequences of more Japanese macaque *B. quintana* strains to clarify whether the same chromosomal inversion is commonly detected among the strains from Japanese macaque.

### Comparison of the virulence genes repertories

The three *B. quintana* strains shared most of the major virulence genes, such as *omp43*, *omp89*, *vompA*, *vompC*, *bafA*, *hbpA*, and *ialB*. However, four important virulence genes (*bepA*, *trwL2*, *trwL4*, and *trwL6*) were missing in the *virB/D4* and *trw* operons of strain MF1-1 (Supplementary Table S4).

**Figure 6:**
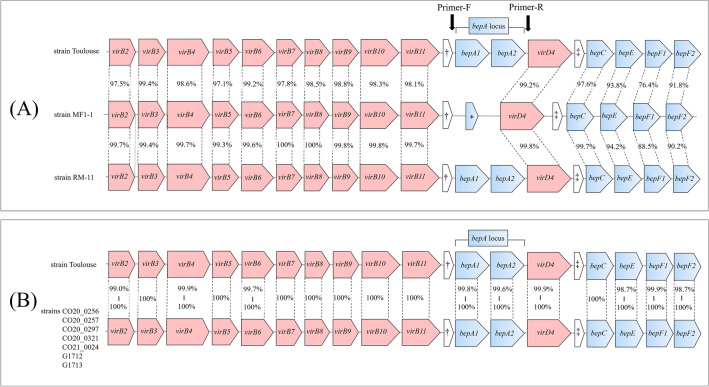
Comparisons of the *virB/D4* operon structure among *B. quintana* strains. (**A**) Comparison of the *virB/D4* operon structures among strains Toulouse, MF1-1, and RM-11. (**B**) Comparison of the *trw* operon structures among strain Toulouse and other human and human louse strains. Orthologous genes are connected by dashed lines, and nucleotide sequence identities are shown for each compared gene. Structural genes of the VirB/D4 T4SS are presented in red and the *bep* genes are presented in light blue. Black arrows indicate primer-binding sites of the *bepA* locus. †, pseudo gene; ‡, function-uknown gene; *, remnant sequence of the *bepA2*.

**Figure 7:**
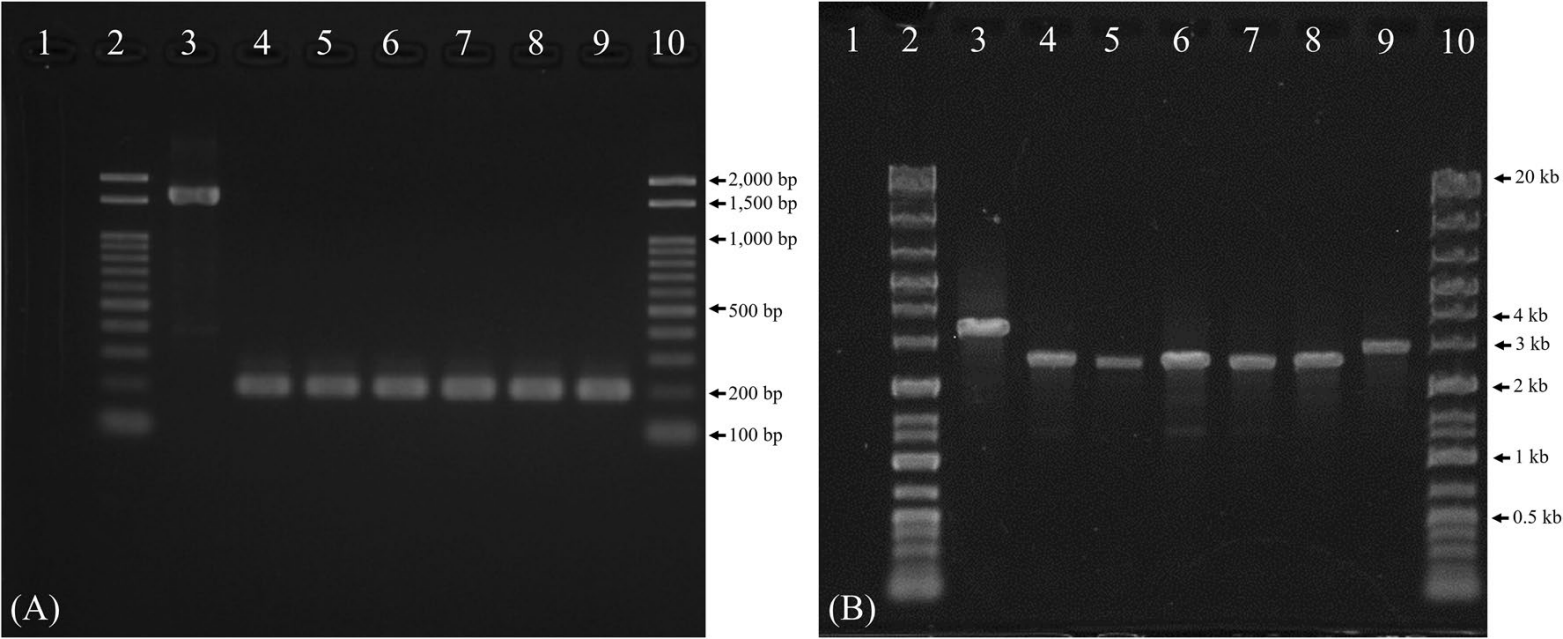
Electrophoresis images of the *bepA*- and the *trwL*-specific PCRs for the Japanese macaque strains. (**A**) An electrophoresis image of the *bepA*-PCR products. (**B**) An electrophoresis image of the *trwL*-PCR products. Lane 1, nuclease-free water for negative control; Lanes 2 and 10, DNA ladder marker; Lane 3, *B. quintana* strain Fuller^T^ DNA for positive control; Lane 4, strain MF1-1; Lane 5, strain MF3-1; Lane 6, strain MF10-1; Lane 7, strain MF11-1; Lane 8, strain MF19-1; Lane 9, strain MF34-1. The DNA ladder sizes are 0.1–2 kb for the *bepA*-specific PCR and 0.1–20 kb for the *trwL*-specific PCR.

Highly conserved *virB/D4* operon structures were observed between strain Toulouse and strain RM-11 (Fig. 6A) and between strain Toulouse and the other *B. quintana* strains from humans and human louse (Fig. 6B). Although only a remnant (141 bp) of *bepA2* was observed in strain MF1-1 (Fig. 6A), BID domain was absent in the gene (Supplementary Fig. S4). Interestingly, the deletion of the *bepA* locus was confirmed by PCR and sequencing of the PCR products in all the five other Japanese macaque strains examined (Fig. 7A), suggesting that gene deletion is a common mutation in the *B. quintana* strains of Japanese macaques. The *bepA* genes contribute to developing vascular tumors by exerting an anti-apoptotic function against *B. quintana*-infected endothelial cells^9^. Thus, the tumorigenic ability of Japanese macaque strains may be lower than that of human and rhesus macaque strains. According to the evolutionary biology of macaque monkeys, some of the rhesus macaque-like populations dispersed from the Korean Peninsula to the Japanese archipelago 0.63–0.43 million years ago, then an independent species (i.e., Japanese macaque) emerged^43^. Considering the present results and the evolutionary history of both macaque species, it is likely that *bepA1* and *bepA2* were lost from the *B. quintana* genome during host divergence from the rhesus macaque to the Japanese macaque.

**Figure 8:**
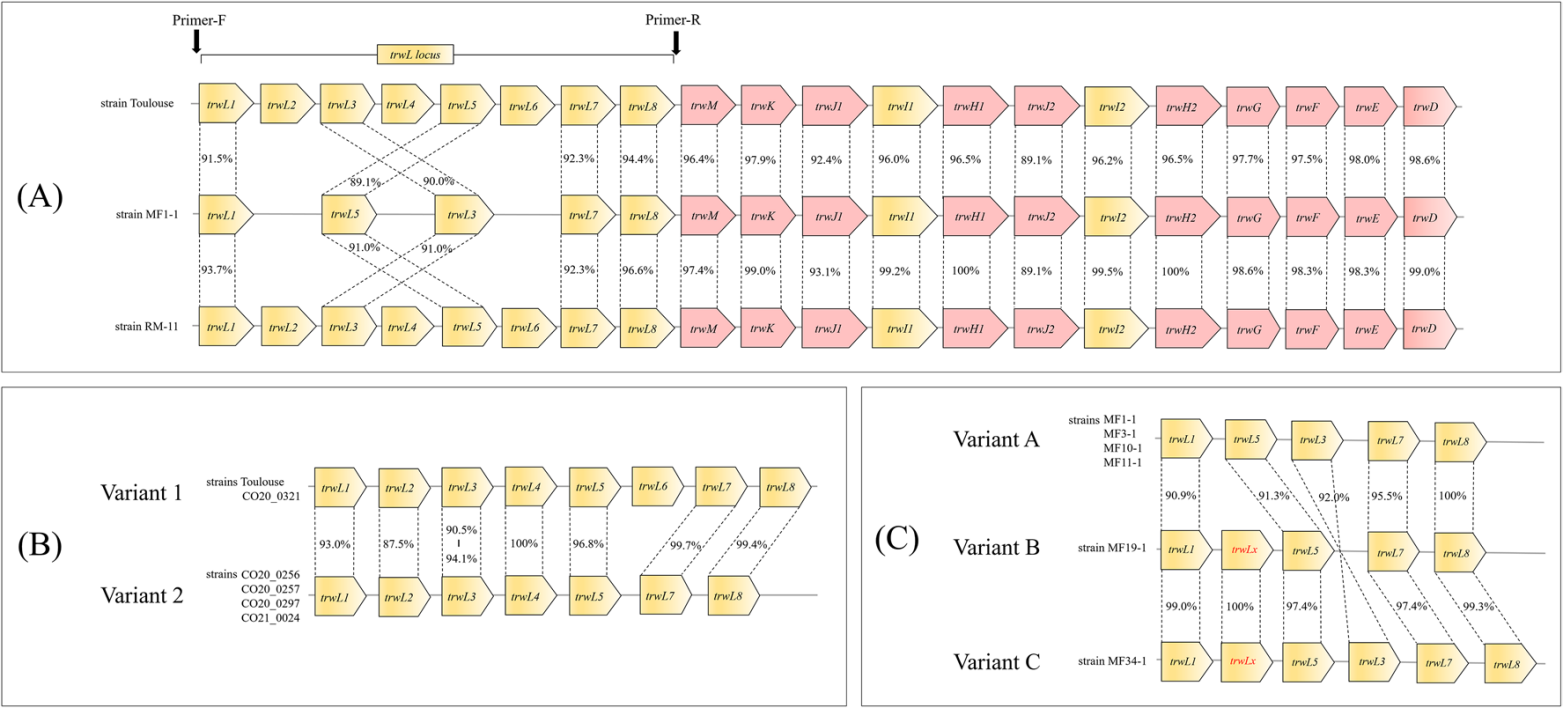
Comparisons of the *trw* operon structure among *B. quintana* strains. (**A**) Comparison of the *trw* operon structure among strains Toulouse, MF1-1, and RM-11. (**B**) Structural variants of the *trwL* locus identified in human strains. (**C**) Structural variants of the *trwL* locus identified in Japanese macaque strains. Orthologous genes are connected by dashed lines, and nucleotide sequence identities are shown for each compared gene. Genes encoding major or minor pili in the Trw T4SS are presented in yellow, and the other genes-related to the Trw T4SS are in red. Black arrows indicate primer-binding sites of the *trwL* locus.

Strains Toulouse and RM-11 had highly conserved *trw* operon structures (Fig. 8A). Based on this result, we analyzed in silico the gene copy numbers of the *trwL* locus in the other sequenced human strains. Strain CO20_0321 showed the same variant (structural variant 1) as strain Toulouse, and strains CO20_0256, CO20_0257, CO20_0297, and CO21_0024 were classified into another variant (structural variant 2) with the absence of the *trwL6* (Fig. 8B). The remaining strains G1712 and G1713 could not be classified due to ambiguous sequences of the locus. These results indicate that *B. quintana* strains from humans have at least two structural variants of the *trwL* locus. In contrast, strain MF1-1 was found to lack the *trwL2*, *trwL4*, and *trwL6* in the locus. The *trwL* genes of strain MF1-1 were tandemly aligned in the order of L1-L5-L3-L7-L8 (structural variant A), which remarkably differed from the gene orders of strains RM-11 and Toulouse. When the *trwL* locus was examined in the other five Japanese macaque strains, structural variant A was detected in strains MF3-1, MF10-1, and MF11-1, as in strain MF1-1, and structural variants B and C were newly identified in strains MF19-1 and MF34-1, respectively (Figs. 7B and 8C). Moreover, an untypable *trwL* (*trwLx*) was found between *trwL1* and *trwL5* in structural variants B and C. When performing pairwise comparison for the *trwL* genes in structural variants B and C, the nucleotide identities to the *trwLx* ranged from 57.4% (vs. *trwL8*) to 80.7% (vs. *trwL1*) in variant B and from 57.8% (vs. *trwL8*) to 89.8% (vs. *trwL3*) in variant C (Supplementary Fig. S5). Subsequently, when the *trwLx* was compared with the *trwL2*, *trwL4*, and *trwL6* in variants 1 and 2 of the human strains, the nucleotide identities ranged from 75.6% (vs. *trwL2*) to 79.0% (vs. *trwL6*) in variant 1 and from 77.3% (vs. *trwL4*) to 78.6% (vs. *trwL2*) in variant 2 (Supplementary Fig. S6). These results support that *trwLx* may be specific in the *trwL* gene of Japanese macaque strains.

The *trwL* locus of *Bartonella* species is presumed to have evolved by repetitive gene duplication^12^; therefore, the genomic structure of the *trwL* locus may have drastically altered. It has been thought that the *B. quintana* genome contains eight copies of the *trwL* in the operon with the order L1-L2-L3-L4-L5-L6-L7-L8^12^. However, it must be insufficient to identify the genomic structure of the locus, because this knowledge is derived from a study using only strain Toulouse. The present study identified that there are several structural variants of the *trwL* locus in the *B. quintana* strains from humans and Japanese macaques. To clarify the relationship between the *trwL* variants and host specificity for *B. quintana*, we need to elucidate more details of variations in other *B. quintana* strains from Japanese macaques as well as other hosts.

### Supplementary Information

Supplementary Information 1.

Supplementary Information 2.

Supplementary Information 3.

Supplementary Information 4.

Supplementary Information 5.

## Data Availability

The genome sequence data of *B. quintana* strain MF1-1 have been deposited in the INSD via the DDBJ center and have been assigned accession number AP019773. These data have also been assigned the RefSeq accession number NZ_AP019773 in the RefSeq database managed by the NCBI. The *trwL* genes of strain MF1-1 (variant A), MF19-1 (variant B), and MF34-1 (variant C) have been deposited in the same database and have been assigned the following accession numbers: LC805960 (*trwL1*), LC805961 (*trwL3*), LC805962 (*trwL5*), LC805963 (*trwL7*), and LC805964 (*trwL8*) in strain MF1-1; LC805965 (*trwL1*), LC805966 (*trwL5*), LC805967 (*trwL7*), LC805968 (*trwL8*), and LC805969 (*trwLx*) in strain MF19-1; LC805970 (*trwL1*), LC805971 (*trwL3*), LC805972 (*trwL5*), LC805973 (*trwL7*), LC805974 (*trwL8*), and LC805975 (*trwLx*) in strain MF34-1.
