# Supplementary material for: Genomic properties of a Bartonella quintana strain from Japanese macaque (Macaca fuscata) revealed by genome comparison with human and rhesus macaque strains
Source: Sci Rep. 2024 May 13;14:10941. doi: 10.1038/s41598-024-61782-0 (PMC11091102; doi:10.1038/s41598-024-61782-0)
Supplement: Supplementary file 1 — Supplementary Information 1. [file 41598_2024_61782_MOESM1_ESM.pdf]

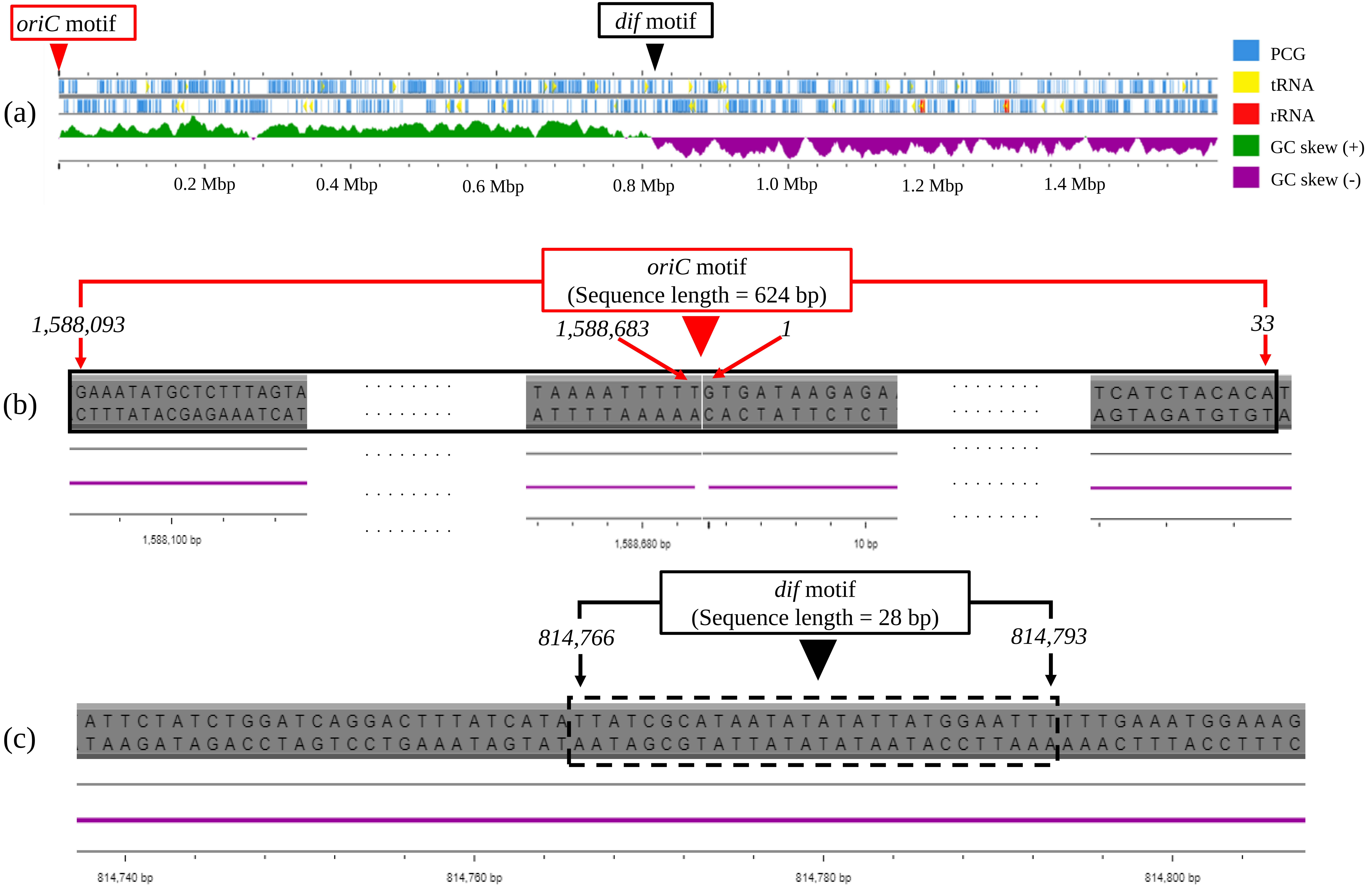

Supplementary Figure S1. Linear genome map of *B. quintana* strain MF1-1.
(a) Forward and reverse strands are the upper line and the lower line, respectively. The red arrowhead indicates a genomic sequence near the replication origin (*oriC*) of strain MF1-1 chromosome, whereas the black arrowhead indicates a genomic sequence near the terminus region (*dif*) of the same strain. (b) Black line square indicates the range of the nucleotide sequence of the *oriC* motif (the putative sequence length = 624bp). (c) Black dashed line indicates the range of the nucleotide sequence of the *dif* motif (the putative sequence length = 28bp).

Host	Country	Strain	MF1-1	RM-11	Toulouse	CO20_0256	CO20_0257	CO20_0297	CO20_0321	CO21_0024	G1712	G1713
Japanese macaque	Japan	MF1-1	100	99.4	98.3	98.3	98.3	98.3	98.3	98.3	98.2	98.2
Rhesus macaque	China	RM-11	99.4	100	98.3	98.3	98.3	98.3	98.3	98.3	98.3	98.3
Human	France	Toulouse	98.3	98.3	100	99.8	99.8	99.8	99.9	99.8	99.5	99.5
Human	USA	CO20_0256	98.3	98.3	99.8	100	99.9	99.9	99.8	99.9	99.5	99.5
Human	USA	CO20_0257	98.3	98.3	99.8	99.9	100	99.9	99.8	99.9	99.5	99.5
Human	USA	CO20_0297	98.3	98.3	99.8	99.9	99.9	100	99.8	99.9	99.5	99.5
Human	USA	CO20_0321	98.3	98.3	99.9	99.8	99.8	99.8	100	99.8	99.5	99.5
Human	USA	CO21_0024	98.3	98.3	99.8	99.9	99.9	99.9	99.8	100	99.5	99.5
Human	Senegal	G1712	98.2	98.3	99.5	99.5	99.5	99.5	99.5	99.5	100	99.9
Human louse	Senegal	G1713	98.2	98.3	99.5	99.5	99.5	99.5	99.5	99.5	99.9	100

Percentage (%)

100
99.5
99.0
98.5
98.0

Supplementary Figure S2. Heat map of the average nucleotide identity values (%) from a pairwise comparison of 10 *B. quintana* strains from a Japanese macaque, a rhesus macaque, humans, and a human louse.

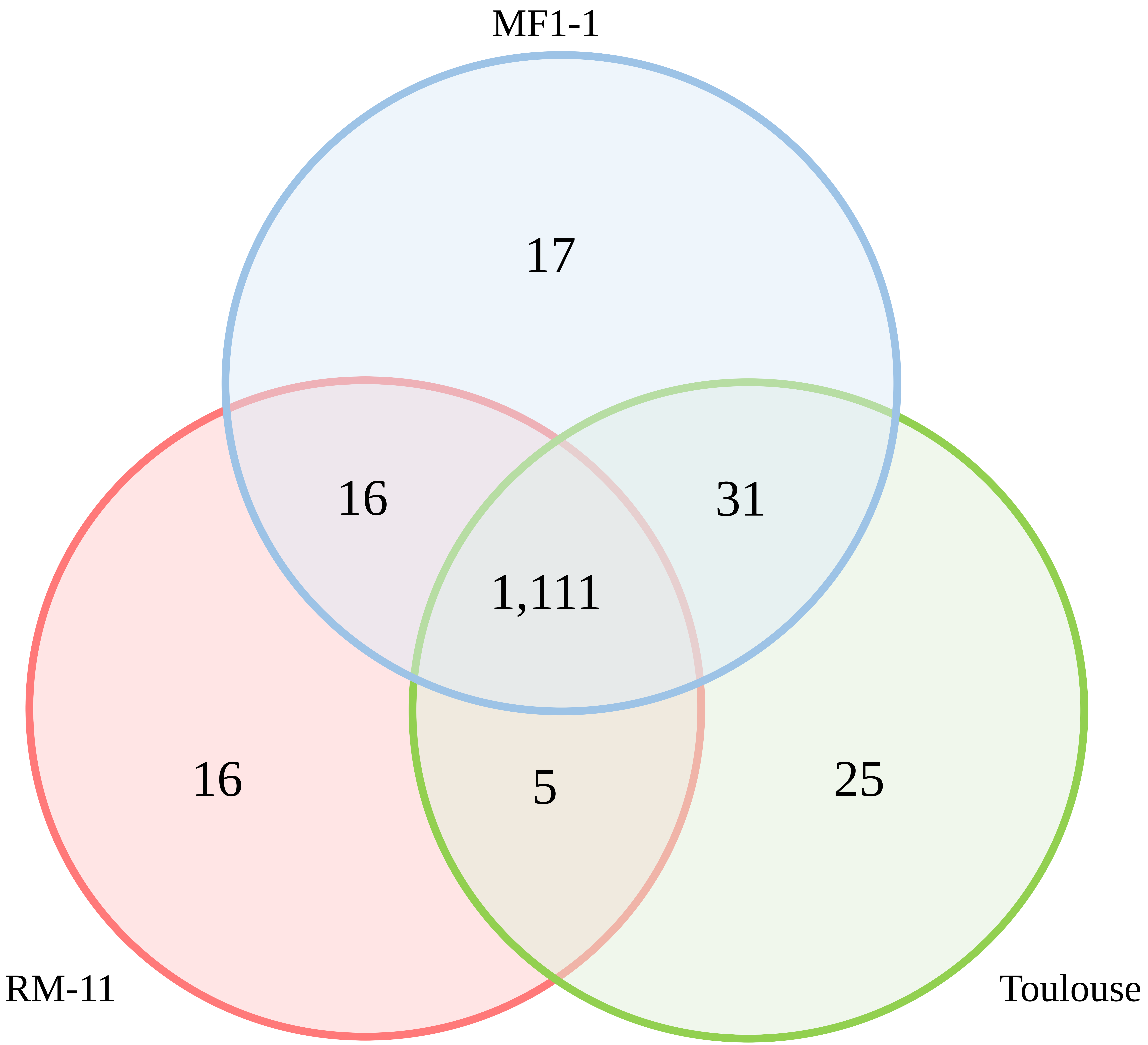

Supplementary Figure S3. Distribution of PCGs of *B. quintana* strains MF1-1, RM-11, and Toulouse.
Colored circles highlight the number of PCGs for three *B. quintana* strains: strain MF1-1 (blue), strain RM-11 (red), and strain Toulouse (green). The number of core-genome genes is shown in the area overlapped by the three circles. To avoid ambiguities due to identical paralogs within one genome, multiple paralogous genes were reduced to a representative gene in this calculation. The core-genome genes and their locus tags are summarized in Supplementary Table S3.

BID domain (411 bp)

```
line 1  CACCTTTACAGCTCCAAGGACCAGAGGCCTTGAAAACACTCTCATTTCAGAAGAAACATTGGCTCCTTTGACAAAAATGGAAATAGCCGAAATGGCTGCA
line 2  CACCTTTACAGCTCCAAGGACCAGAGACCTCGAAAACACTTTCATCTCAAAAGAAACATTGGCTCCTTTGACAAAAATGGAAATGGCCGAAATGGCTGCA
line 3  ----------------------------------------------------------------------------------------------------

line 1  GAAGATGCCCGTGTTCACACATGTCGAGACCAAATTCACAAATTGTCAGAAATTGTTTATGGCAGCTCGAAAACATTGGACCAAAAAATGGTCGAGATTA
line 2  GAAGATGCCCGTGTTCACACATGTCGAGACCAAATTCACAAATTGTCAGAAATTGTTTATGGCAGCTCGAAAACATTGGACCAAAAAATGGTCGAGATTA
line 3  ----------------------------------------------------------------------------------------------------

line 1  TTAAAAATCCAAGCGTGGGTGCCCTGCTTGCCGAACAGATTGAAAAGTCTCCACACTCTCTTGCAAATCTTGCAGGTTTTGACCTCATTTGTTTTAAAAG
line 2  TTAAAAATCCAAGCGTGGGTGACCAGCTTGCCGAACAGATTGAAAAGTCTCCACACTCTCTTGCAAATCTTGCAGGTTTTGACCTCATTTGTTTTAAAAG
line 3  ----------------------------------------------------------------------------------------------------

line 1  TCGGGCCCGGATAAATGCGGAAGAAAATGTTGAACTCCTCGGTTGCGCCGTTTCAAATTTTGCCTTTGCGGTAAGACAGGCTAAGAAAGAAATCACGCAA
line 2  TCGGGCCCGGATAAAGGCGAAAGAAAATGTTGAACTCCTCAGTTGCGCCGTTTCAAATTTTGCCTTTGCGGTAAGACAGGCTAAGAAAGAAATCACGCAA
line 3  ----------------------------------------------------------------------------------------------------

line 1  GAACATCAAGCAGAACAAAACCGCCGTGGACAAGAGGTAGCAATGCCAAGCCAAAGCTTGAAAAATCTCCTTTCCATGCCAAAAGAATTGCAGCAAACAG
line 2  GAACATCAAGCAGAACAAAACCGCCGTGGACAAGAAGTAGCAATGCCAAGCCAAAGCTTGAAAAATCTCCTTTCCATGCCAAAAGAATTGCAGCAAACAG
line 3  ----------------------------------------------------------------------------------------------------

line 1  CTTTAGCAGCTTCTCCTTTGCTTCAAAAAGAGCTCGAAAGTCTTATAAAACAAGTCAGTAACCGTCTCTCAGGAAGCGAGCAAAGAGCCCTCAAGAACAA
line 2  CTTTAGCAGCTTCTCTTTTGCTTCAAAAAGAGCTCGAAAGTCTTATAAAACAAGTCAGTAACCGTCTCTCAGGAAACGAGCAGAGAGCCCTCAAGAACAA
line 3  ----------------------------------------------------------------------------------------------------

line 1  GGATTATGATGCACTCGTGAAAAGTCTTGGTGTCTCACAACATAAAGCAAAGGAAATTACACAGACCGTTAATCAAGCCAGAGAGGCACAGAAACAATTG
line 2  GAATTATGATGCACTCGCGAAAAATCTTGGTGTCTCACAACATAAAGCAAAGGAAATTACACAGACCGTTAATCAAGCCAGAGATGCACAAAAACAATTG
line 3  --------ATGCTAACTTATAAGGAGGTGGTTATGCCAAACA-----CAAAGGAAATTACAGAGACCGTTCA-CAAGCCAGAGAGGCACAAAAACAATTG

line 1  CAAACACAGACAGTCAGTCGCTCAAAAGTGCTCGCCATGGGGCAAGCTAAAATAGTTTGCTAA
line 2  CAAACACGGACAGTCAGTCGCTCAAAAGTGCTCGCCATGGGGCAAGCTAAAATAGTTTGCTAA
line 3  CAAACACGGACAGTCAGTCGCTCAAAAGTGCTCGCCATGGGGCAAGCTAAAATAG--------
```

Remnant of the *bepA2* gene (141 bp)

Supplementary Figure S4. Comparison of the *bepA2* gene among strains Toulouse, RM-11, and MF1-1.
Lines 1, 2, and 3 indicate the C-terminal regions of the *bepA2* genes in strains Toulouse, RM-11, and MF1-1, respectively. Black line square indicates a region of BID domain in strains Toulouse and RM-11 and blue line square indicates a region of remnant of the *bepA2* gene of strain MF1-1.

	trwLx	*trwL1*	*trwL5*	*trwL7*	*trwL8*
trwLx	100	80.7	77.0	77.2	57.4
trwL1	80.7	100	73.7	73.1	56.1
trwL5	77.0	73.7	100	82.6	56.5
trwL7	77.2	73.1	82.6	100	56.7
trwL8	57.4	56.1	56.5	56.7	100

A pairwise comparison of the *trwL* genes in variant B

Percentage (%)
100
90.0
80.0
70.0
60.0
50.0

	trwLx	*trwL1*	*trwL3*	*trwL5*	*trwL7*	*trwL8*
trwLx	100	80.4	89.8	76.9	76.9	57.8
trwL1	80.4	100	80.4	74.5	72.6	56.2
trwL3	89.8	80.4	100	74.6	78.7	56.6
trwL5	76.9	74.5	74.6	100	79.6	56.3
trwL7	76.9	72.6	78.7	79.6	100	56.9
trwL8	57.8	56.2	56.6	56.3	56.9	100

A pairwise comparison of the *trwL* genes in variant C

Supplementary Figure S5. Heat map of the nucleotide identity values (%) from a pairwise comparison of the *trwL* genes in variants B and C. Upper and lower panels indicate results of pairwise comparisons of the *trwL* genes in variants B and C, respectively.

	Variant 1			Variant 2	
	trwL2	*trwL4*	*trwL6*	*trwL2*	*trwL4*
trwLx	75.6	77.3	79.0	78.6	77.3

Supplementary Figure S6. The nucleotide identity values (%) of the *trwLx* to the *trwL* genes in variants 1 and 2.
