# Supplementary material for: Genomic properties of a Bartonella quintana strain from Japanese macaque (Macaca fuscata) revealed by genome comparison with human and rhesus macaque strains
Source: Sci Rep. 2024 May 13;14:10941. doi: 10.1038/s41598-024-61782-0 (PMC11091102; doi:10.1038/s41598-024-61782-0)
Supplement: Supplementary file 2 — Supplementary Information 2. [file 41598_2024_61782_MOESM2_ESM.docx]

Supplementary Table S1. Primers for *bepA*- and *trwL*-specific PCRs developed in the present study.

| Targeted loci | Primer names | Nucleotide sequences | Use application |
| --- | --- | --- | --- |
| *bepA* locus | bepA_f | 5’-TAACACGCTTATGGACAATG-3’ | PCR and sequencing |
|  | bepA_r | 5’-AGTCAAAGGAAGGTGCAA-3’ | PCR and sequencing |
|  | iF_Munich | 5’-CATAGAAGGCAATGAGCGC-3’ | Sequencing |
|  | iR_Mun | 5’-TTTCAATCTGTTCGGCAAGCC-3’ | Sequencing |
|  | overR | 5’-CTGGCATGACAGCAGTGG-3’ | Sequencing |
|  | overF | 5’-ACAAGGATTATGATGCACTCG-3’ | Sequencing |
| *trwL* locus | MF1_RS06065-Fow1 | 5'-AAGCAGTGTTCGTGATGCTT-3' | PCR and sequencing |
|  | MF1_RS06095-Rev1 | 5'-TAACTATAAGCGGCATCATT-3' | PCR and sequencing |
|  | inner01-Rev | 5'-TTCACTTTCATTATCCAG-3' | Sequencing |
|  | inner01-Fow | 5'-CATGATGAAAATGAAAAACA-3' | Sequencing |
|  | trwL1-Fow1 | 5'-CTGCATAGAAAGCTTTTCA-3' | Sequencing |
|  | trwL1-R1 | 5'-ATGTTTTTCATTTTCATCATG-3' | Sequencing |
|  | inner-pr1 | 5'-TACAATCATAGCTGCGATA-3' | Sequencing |
|  | inner-pr2 | 5'-TATCGCAGCTATGATTGTA-3' | Sequencing |
