# Supplementary material for: Genomic properties of a Bartonella quintana strain from Japanese macaque (Macaca fuscata) revealed by genome comparison with human and rhesus macaque strains
Source: Sci Rep. 2024 May 13;14:10941. doi: 10.1038/s41598-024-61782-0 (PMC11091102; doi:10.1038/s41598-024-61782-0)
Supplement: Supplementary file 3 — Supplementary Information 3. [file 41598_2024_61782_MOESM3_ESM.docx]

| Targeted loci  (Expected amplicon size) | PCR steps (# of cycles) | | Temperature | Time |
| --- | --- | --- | --- | --- |
| *bepA* locus  (1,612 bp) | Pre-denaturation (×1) | | 94℃ | 5 min |
|  | Amplification (×35) | Denaturation | 94℃ | 20 sec |
|  |  | Annealing | 56℃ | 30 sec |
|  |  | Extension | 72℃ | 30 min |
|  | Final extension (×1) | | 72℃ | 5 min |
| *trwL* locus  (4,026 bp) | Pre-denaturation (×1) | | 94℃ | 2 min |
|  | Amplification (×30) | Denaturation | 94℃ | 20 sec |
|  |  | Annealing | 58℃ | 30 sec |
|  |  | Extension | 72℃ | 30 sec |
|  | Final extension (×1) | | 72℃ | 5 min |

Supplementary Table S2. Conditions for *bepA*- and *trwL*-specific PCRs developed in the present study.

The amplicon sizes in both loci were estimated by *in silico* calculation based on the complete genome sequence of strain Toulouse (RefSeq #: NC_005955).
